# Deafening Silence of Malaysian Medical and Surgical Fraternities to the Gaza Genocide

**DOI:** 10.5704/MOJ.2407.015

**Published:** 2024-07

**Authors:** ZF Zairul-Nizam, NA Ibrahim

**Affiliations:** 1 Department of Surgery, International Medical University, Seremban, Malaysia; 2 Department of Anaesthesiology and Intensive Care, Universiti Putra Malaysia, Serdang, Malaysia

Dear editor,

Dr Adnan al-Bursh was head of orthopaedics at al-Shifa Hospital, the largest medical facility in the Gaza Strip. Some have anecdotally called him the best orthopaedic surgeon in the land. When hostilities knocked the front door of al-Shifa hospital, instead of heading south as ‘requested’ by the Israeli Occupation Force (IOF), he went north to continue serving his people until his arrest in December 2023. On 19 April 2024, news reached the world that Dr al-Bursh died in prison, likely from torture^1^.

Dr Mehdat Sedim^2^ was one of the few board-certified plastic and burn surgeons in Gaza. Along with the Dean of the Islamic University of Gaza School of Medicine, Dr Omar Ferwana^3^, Dr Sedim was killed in October 2023. The United Nations, on 16 May 2024, expressed ‘concern’ that since 7 October 2023, at least 493 doctors have been killed in the line of duty^4^. By now, the number might well be at least 500 and the above are merely three from the long list of Gazan doctors fallen.

As volunteers to Rafah, we (corresponding author was in Mercy Malaysia Medical Mission Team 2, 16 March – 03 April 2024) have seen first-hand the carnage inflicted there. We have heard accounts of medical personnel and facilities being targeted and systematically annihilated. How civilians bring clothes for doctors and nurses to change from their scrubs, lest they be victimised. With the Gazan healthcare system in shambles, the continued killings and abductions of healthcare personnel can only exponentially worsen an already dire situation.

The question remains why a united voice is hardly raised against these atrocities by Malaysia doctors. Those doctors and nurses, technicians and therapists, are our colleagues and brethren, despite the miles that separate us. Their plight should be our own. They deserve our unified and unrelenting support, not relegated to hushed undertones of pitiful condemnation against the oppressors in small circles.

We cannot be so dispassionate as to view this simply as ‘a political matter outside the realm of medicine’ as one senior editor puts it. We are bound, if not by our sacred medical oath, then by our consciousness and humanity, to preserve the sanctity of human life whenever and with whatever means possible^5^. When we left Rafah, those we left behind implored us to tell the world of their plight. What better platform than the combined voice of so many medical and surgical fraternities. Yet, more than 9 months and 38,000 deaths later, that voice is limited to a few webinars and small-group presentations.

It is utterly disheartening that we, as a society, did not openly condemn the assassination of our colleagues and this genocidal madness. What could possibly have prevented us from speaking truth to power? We cannot allow ourselves to be so immersed in our own comfort that another person’s troubles remain just that: theirs, not ours. That would amount to betraying the memory of Dr al-Bursh and Dr Sedim and all the other martyrs. How shameful that would be.

Plato wrote that ‘silence is consenting’. At this point, through our continued silence, it appears as though we are.
